# Development of a human vasopressin V_1a_-receptor antagonist from an evolutionary-related insect neuropeptide

**DOI:** 10.1038/srep41002

**Published:** 2017-02-01

**Authors:** Maria Giulia Di Giglio, Markus Muttenthaler, Kasper Harpsøe, Zita Liutkeviciute, Peter Keov, Thomas Eder, Thomas Rattei, Sarah Arrowsmith, Susan Wray, Ales Marek, Tomas Elbert, Paul F. Alewood, David E. Gloriam, Christian W. Gruber

**Affiliations:** 1 Center for Physiology and Pharmacology, Medical University of Vienna, Schwarzspanierstrasse 17, 1090 Vienna, Austria; 2 Institute for Molecular Bioscience, The University of Queensland, QLD 4072 Brisbane, Australia; 3 Department of Drug Design and Pharmacology, University of Copenhagen, Jagtvej 162, 2100 Copenhagen, Denmark; 4 School of Biomedical Sciences, The University of Queensland, QLD 4072 Brisbane, Australia; 5 IST Austria (Institute of Science and Technology), Am Campus 1, 3400 Klosterneuburg, Austria; 6 CUBE-Division of Computational Systems Biology, Department of Microbiology and Ecosystem Science, University of Vienna, Althanstrasse 14, 1090 Vienna, Austria; 7 Harris-Wellbeing Preterm Birth Research Centre, Department of Cellular and Molecular Physiology, Institute of Translational Medicine, University of Liverpool, L69 3BX, United Kingdom; 8 Laboratory of Radioisotopes, Institute of Organic Chemistry and Biochemistry CAS, Flemingovo nám. 2, CZ-16610 Prague 6, Czech Republic

## Abstract

Characterisation of G protein-coupled receptors (GPCR) relies on the availability of a toolbox of ligands that selectively modulate different functional states of the receptors. To uncover such molecules, we explored a unique strategy for ligand discovery that takes advantage of the evolutionary conservation of the 600-million-year-old oxytocin/vasopressin signalling system. We isolated the insect oxytocin/vasopressin orthologue inotocin from the black garden ant (*Lasius niger*), identified and cloned its cognate receptor and determined its pharmacological properties on the insect and human oxytocin/vasopressin receptors. Subsequently, we identified a functional dichotomy: inotocin activated the insect inotocin and the human vasopressin V_1b_ receptors, but inhibited the human V_1a_R. Replacement of Arg8 of inotocin by D-Arg8 led to a potent, stable and competitive V_1a_R-antagonist ([D-Arg8]-inotocin) with a 3,000-fold binding selectivity for the human V_1a_R over the other three subtypes, OTR, V_1b_R and V_2_R. The Arg8/D-Arg8 ligand-pair was further investigated to gain novel insights into the oxytocin/vasopressin peptide-receptor interaction, which led to the identification of key residues of the receptors that are important for ligand functionality and selectivity. These observations could play an important role for development of oxytocin/vasopressin receptor modulators that would enable clear distinction of the physiological and pathological responses of the individual receptor subtypes.

G protein-coupled receptors (GPCRs) represent one of the largest families of membrane bound proteins in the human genome^1^,^2^. They form attractive drug targets as they play a crucial role in the regulation of physiological signalling systems and are accessible at the cell surface^3^. In recent years, the field of GPCR research has evolved at great pace; advances in structural characterisation of several GPCRs has led to detailed analyses of their pharmacological properties^4^,^5^. Such studies rely heavily on the availability of a chemical toolbox of compounds^6^ that selectively stabilize conformational states of GPCRs. The alliance between chemistry and pharmacology has led to astonishing breakthroughs in understanding ligand-receptor interactions and the development of valuable therapeutics.

The oxytocin/vasopressin signalling system constitutes one of the most complex and important neuroendocrine systems in humans. There are four known receptor subtypes, the oxytocin receptor (OTR) and vasopressin receptor isoforms V_1a_R, V_1b_R and V_2_R^7^. These receptors belong to the largest subclass of the rhodopsin-β adrenergic receptor family (class A)^8^ and are activated by the two endogenous peptide ligands oxytocin and arginine-vasopressin. Despite the lack of a crystal structure, several strategies have attempted to identify and develop receptor probes to study the structure-activity relationships and molecular signalling properties of these GPCRs (summarized in refs 9,10). Nonetheless, the structural homology of endogenous peptides and receptors is a major inconvenience and hampers the development of powerful molecular probes^9^. Although the oxytocin/vasopressin signalling system is linked to a diverse range of high-profile disorders including preterm labour, cancer, pain, autism, anxiety, stress, depression, and cardiovascular diseases^7^,^11^,^12^, there is still a lack of selective receptor agonists and antagonists, which limits our ability to characterize the physiological and pathological function of each receptor subtype^9^,^13^.

In humans and other mammalian species, oxytocin and vasopressin signalling displays an extensive repertoire of peripheral physiological functions, including uterine smooth muscle contraction during parturition, ejaculation, milk let-down, vasoconstriction and osmoregulation (summarized in ref. 14). Centrally, this neuropeptidergic system has been implicated in memory and learning, social cognition and aggressive behaviour^15^,^16^,^17^,^18^. Oxytocin (CYIQNCPLG-NH_2_) and arginine-vasopressin (CYFQNCPRG-NH_2_) are cyclic nonapeptides containing a disulfide bond between Cys-residues in positions 1 and 6, differing only in two positions in most mammals (residues 3 and 8)^9^. Oxytocin- and vasopressin-like peptides have been identified in all vertebrate animals, as well as in some invertebrate species^19^. These peptides are evolutionarily highly conserved and functionally related^19^,^20^. The arthropod orthologue of oxytocin and vasopressin, called inotocin (CLITNCPRG-NH_2_)^21^,^22^, also exhibits a high degree of structural conservation offering a unique opportunity to probe structure/function-relationships of the human oxytocin and vasopressin receptors to increase our molecular understanding of these GPCRs^23^.

In the present study, we isolated, cloned and characterized the inotocin receptor with its cognate ligand inotocin from the black garden ant (*Lasius niger*) and exploited its evolutionary relationship and structural similarity to the human vasopressin V_1a_ and V_1b_ receptors. Our approach aimed at demonstrating that exploring a highly conserved and widely distributed signalling system such as the oxytocin/vasopressin system is a good strategy for the discovery and development of much needed selective probes for the study of the four human receptor subtypes. To validate the pharmacological tools, we determined the molecular properties of the endogenous ant ligand-receptor pair, in comparison to the previously identified inotocin receptor of the red flour beetle (*Tribolium castaneum*). Based on the molecular conservation of the insect and human signalling systems, we conducted *in vitro* pharmacological studies of the insect inotocin at the four human oxytocin/vasopressin receptors. Due to the observed binding and activation profiles of the insect ligand, we probed the structure-activity relationship by replacing Arg8 of inotocin with its corresponding D-stereoisomer to investigate how this amino acid substitution influences ligand binding and selectivity. This approach successfully yielded a potent, selective and very stable antagonist of the human V_1a_R, which was validated in functionally-relevant *ex vivo* uterine contraction studies. These probes furthermore provided novel insights into the molecular binding features and evolution of the ligand-receptor pairs of the oxytocin/vasopressin signalling system.

## Results

### Transcriptome analysis and discovery of the inotocin pre-prohormone and partial receptor sequences from *L. niger*

**Figure 1:**
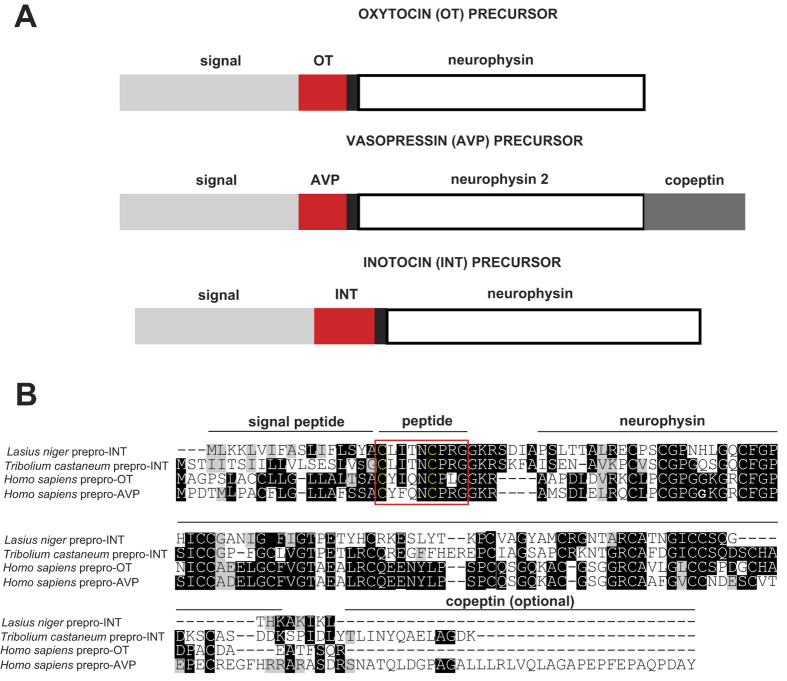
Neuropeptide prepro-hormones of humans and insects. (**A**) Oxytocin (OT), arginine-vasopressin (AVP) and inotocin (INT) peptide precursors share a similar structure with signal peptide (grey), GKR processing signal (black), neurophysin domain (white) and copeptin (dark grey, in the case of vasopressin). (**B**) Multiple sequence alignment of the precursor sequences of the black garden ant (*L. niger*) and the red flour beetle (*T. castaneum*) were compared to human oxytocin and vasopressin. Alignment was performed with Clustal Omega and homologous regions were highlighted in Boxshade representation. The mature peptide domains are coloured in red.

As previously published, insect neuropeptides have been discovered by genome-mining^21^, but it is challenging to derive full-length receptor sequences due to the size of the coding sequences and the presence of multiple introns. Therefore, we aimed to identify the inotocin precursor and its receptor in *L. niger* by transcriptome analysis. To reconstruct the transcriptome, we sequenced *L. niger* cDNA *via* paired end Illumina HiSeq 2000 Technology. The initially obtained 85,112,874 raw sequence read pairs were quality and length filtered and the remaining 90.1% high quality pairs were screened for rRNA reads, which led to the removal of 6.8% of the sequence read pairs. Hence, *de-novo* assembly for the reconstruction of the *L. niger* transcriptome was initiated with 71,435,903 of high quality mRNA read pairs. This resulted in 564.8 megabases distributed over 380,930 contigs with a N50 of 3,308 base pairs (bp). These contigs represent different *L. niger* transcripts and isoforms. We identified 99.6% of a defined set of highly conserved genes. This near complete transcriptome was subject to a sequence similarity search using several insect inotocin precursor and receptor sequences as queries. It resulted in 125 hits for the receptor and two significant hits for the precursor namely the contigs comp45093_c0_seq1 and comp45093_c0_seq2. These sequences represent the transcribed sequences of the inotocin precursor and receptor of *L. niger*. The top precursor hit exhibited a similar domain structure as compared to the human proteins (Fig. 1A). The second precursor hit was discarded as it was considered an artefact. The 125 receptor hits represent different isoforms and assembly artefacts of the most homologous receptors in the assembly. To verify that we have identified the inotocin receptor of *L. niger,* we reconstructed the phylogenetic relationship of the most relevant hits (multiple near identical contigs with different length per main hit). The resulting phylogenetic tree confirmed that we indeed detected the inotocin receptor of *L. niger* (Supplementary Fig. 1). The verified receptor and precursor sequences were used as templates for molecular cloning.

### Molecular cloning and characterization of the inotocin signalling system

**Figure 2:**
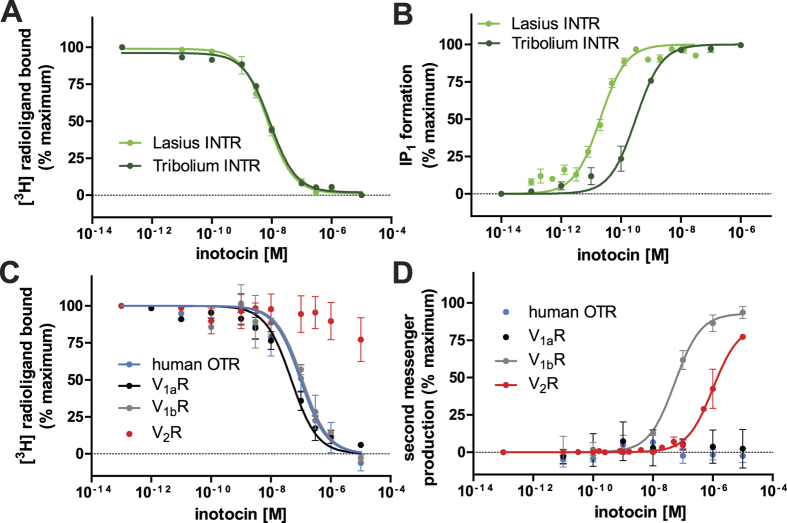
Receptor pharmacology of inotocin on inotocin and human oxytocin/vasopressin receptors. (**A**) Concentration-dependent displacement of [^3^H] inotocin by inotocin at inotocin receptors (INTR) from *Lasius niger* (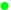) and *Tribolium castaneum* (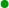) (n = 4). (**B**) Concentration-dependent accumulation of intracellular IP_1_ by inotocin at INTR from *L. niger* (n = 9) and *T. castaneum* (n = 4). (**C**) Concentration-dependent displacement binding of inotocin on human OTR (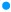), V_1a_R (•), V_1b_R (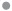) and V_2_R (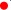) (n = 4). (**D**) Concentration-dependent second messenger production by inotocin at human OTR, V_1a_R, V_1b_R and V_2_R (n ≥ 3). Specific binding was calculated by subtracting the non-specific from the total bound and normalised to 100%. A full description of radioligand and membrane concentrations is reported in the Methods section. Receptor activation was measured by IP_1_ assays for the G_q_-coupled receptors (human OTR, V_1a_R, V_1b_R) and luciferase reporter assay with specific CRE response element for the G_s_-coupled human V_2_R, as described in Methods. Each data point was normalized to percentage of maximal activation, detected at the highest endogenous ligand concentration, being inotocin for inotocin receptor, vasopressin for human V_1a_R, V_1b_R, V_2_R and oxytocin for human OTR. Data is shown as mean ± SEM and fitted by nonlinear regression (sigmoidal, three-parameters, Hill slope of 1).

The full length amplification product corresponding to the novel ant precursor was directly sequenced, and the full length amplification product corresponding to the novel ant receptor was cloned into a plasmid and its sequence confirmed by DNA sequencing. Multiple sequence alignments of precursor (Fig. 1B) and receptor (Supplementary Fig. 2) provided further evidence of the molecular similarity of insect and human oxytocin/vasopressin-like receptors. For functional characterization of the inotocin peptide-receptor system, the peptide inotocin was chemically synthesized and the cDNA encoding the *L. niger* inotocin receptor was cloned into a pEGFP plasmid for transient expression in mammalian cells. For pharmacological validation of the ant signalling system we determined affinity and potency of inotocin to its cognate receptor from *L. niger* and compared the data to the known ligand-receptor pair from *T. castaneum*. The affinity for the inotocin peptides was determined *via* competitive radioligand binding experiments (Fig. 2A) on membrane preparations of mammalian cells transiently expressing these receptors. Inotocin displaced its tritiated analogue with a *K*_i_ of 6 and 10 nM at the ant and beetle receptors, respectively (Table 1). The functional G_q_-coupled receptor response was monitored by detection of the production and accumulation of inositol monophosphate (IP_1_), a downstream metabolite of D-myo-inositol 1,4,5-trisphosphate (IP_3_), following the activation of phospholipase C (Fig. 2B). The endogenous ligand inotocin is able to activate the ant receptor at low picomolar concentrations with an *EC*_50_ value of 22 pM, and the beetle receptor was activated with a potency of 350 pM (Table 2), in agreement with previous published data^24^.

### Pharmacological characterization of inotocin at the human oxytocin and vasopressin receptors

Based on the structural homology of the inotocin peptide, we pursued the pharmacological characterization of the endogenous inotocin on human OTR, V_1a_R, V_1b_R and V_2_R receptors. We determined the affinity of inotocin peptide to the human receptors (Fig. 2C and Table 1) and estimated the ability of this insect peptide to elicit a concentration-dependent functional response (Fig. 2D and Table 2). Inotocin exhibited nanomolar affinity to the human V_1a_R (*K*_i_ = 11 nM), V_1b_R (*K*_i_ = 12 nM) and OTR (*K*_i_ = 62 nM), but does not appear to displace [^3^H] vasopressin from the human V_2_R at concentrations <10 μM (Table 1). Interestingly, the insect ligand is a full agonist at the human V_1b_R (*EC*_50_ = 56 nM), but does not activate the V_1a_R or OTR at a detectable level (*EC*_50_ > 10 μM) (Table 2). Despite being unable to displace [^3^H] vasopressin at the human V_2_R (Table 1), inotocin exhibited partial activation of this receptor with an *EC*_50_ > 1.5 μM, possibly through direct allosteric agonism.

### Pharmacological characterization of the modified ligand [D-Arg8]-inotocin at the human oxytocin and vasopressin receptors

Previous studies indicated that the Arg in position 8 of vasopressin mediates ligand functionality and receptor selectivity in the human oxytocin/vasopressin family^25^,^26^,^27^,^28^. Additionally, the proline-arginine site is easily cleaved by enzymes such as trypsin^29^ and forms a well-known position to improve metabolic stability through introducing D-Arg instead of L-Arg^30^. Having in mind harnessing the insect system to guide the development of novel and stable probes to study human GPCRs, we synthesised [D-Arg8]-inotocin and characterized it pharmacologically at the insect and human receptors (Tables 1 and 2). [D-Arg8]-inotocin is a partial agonist (apparent maximum response *E*_max_ ~80%) at the inotocin receptors from *L. niger* and *T. castaneum* with a potency (*EC*_50_) of 1.2 and 12 nM, respectively, and an affinity (*K*_i_) of 61 and 217 nM, respectively (Supplementary Fig. 3). Interestingly, the D-Arg8 modification resulted in a selectivity profile switch at the human receptor subtypes; [D-Arg8]-inotocin improved its binding affinity at human V_1a_R compared to inotocin (*K*_i_ = 1.3 nM; Table 2 and Supplementary Fig. 3), but did not exhibit any significant displacement at the human OT, V_1b_, V_2_ receptor isoforms (*K*_i_ > 4 μM) (Table 2 and Supplementary Fig. 3). [D-Arg8]-inotocin was not able to evoke a functional IP_1_ response at the human OT, V_1a_ or V_1b_ receptors (*EC*_50_ > 10 μM), yet retained a low potency response at human V_2_R (*EC*_50_ of 164 nM) possibly by direct allosteric agonism as observed with inotocin (Table 2 and Supplementary Fig. 3).

### Competitive antagonism of [D-Arg8]-inotocin at the human V_1a_R

**Figure 3:**
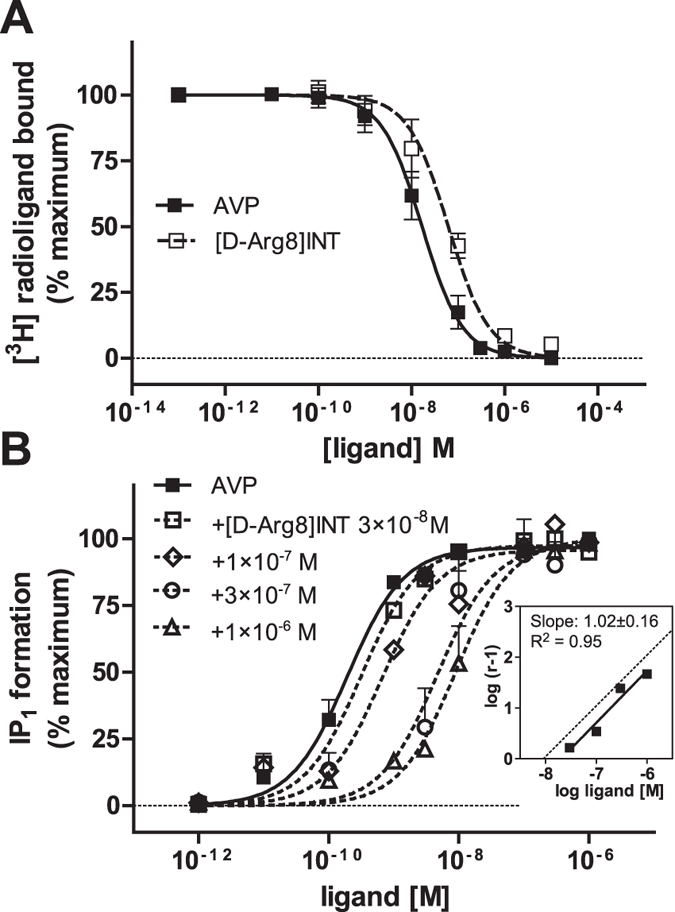
[D-Arg8]-inotocin is a competitive antagonist at the human V_1a_ receptor. (**A**) Concentration-dependent displacement of [^3^H] vasopressin in membrane preparations expressing the human V_1a_R by either [D-Arg8]-inotocin or vasopressin (n = 4). Specific binding was calculated by subtraction of non-specific binding from total binding and normalized to the percentage (%) of maximal binding. (**B**) Concentration-response curves of vasopressin at the human V_1a_R in the absence and presence of varying concentrations of [D-Arg8]-inotocin (30, 100, 300 and 1000 nM). Fold induction of intracellular IP_1_ accumulation above baseline was normalized to the number of cells. Data were fitted by nonlinear regression (sigmoidal, slope = 1). Affinity constants (*K*_i_) and potency (*EC*_50_) values for AVP and [D-Arg8]-inotocin are presented in Tables 1 and 2. Schild regression analysis of [D-Arg8]-inotocin at the human V_1a_R is shown as insert. The corresponding Schild plot is presented in comparison to a linear curve with a slope of 1.0 and the X-intercept of *K*_i_ for [D-Arg8]-inotocin. The pA2 value of 7.8 was derived as X-intercept of the Schild regression curve.

To investigate the inhibitory mechanism of [D-Arg8]-inotocin further, we quantified the concentration-dependent activation of the receptor by the endogenous ligand vasopressin in the absence and presence of a set of constant concentrations of [D-Arg8]-inotocin (30, 100, 300 and 1000 nM, respectively). Typical for a competitive antagonist, we observed a dextral displacement of the concentration-response curves of vasopressin, with no change in the *E*_max_ (Fig. 3B). To confirm a competitive antagonist action of [D-Arg8]-inotocin at the human V_1a_R, we performed Schild regression analysis. The regression of the concentration-ratio, which is a measure of the potency of a drug and the concentration of antagonist [D-Arg8]-inotocin was linear and had a slope of unity, indicating that the antagonism is indeed competitive. [D-Arg8]-inotocin is a competitive inhibitor of the human V_1a_R with a gain of approximately three orders of magnitude selectivity in affinity over the other oxytocin/vasopressin receptors. The pA2 value of this competitive antagonism has been calculated as 7.8 (Fig. 3B), corresponding to a functional affinity of approximately 16 nM.

### Identification of receptor residues responsible for the pharmacological profile of [D-Arg8]-inotocin

**Figure 4:**
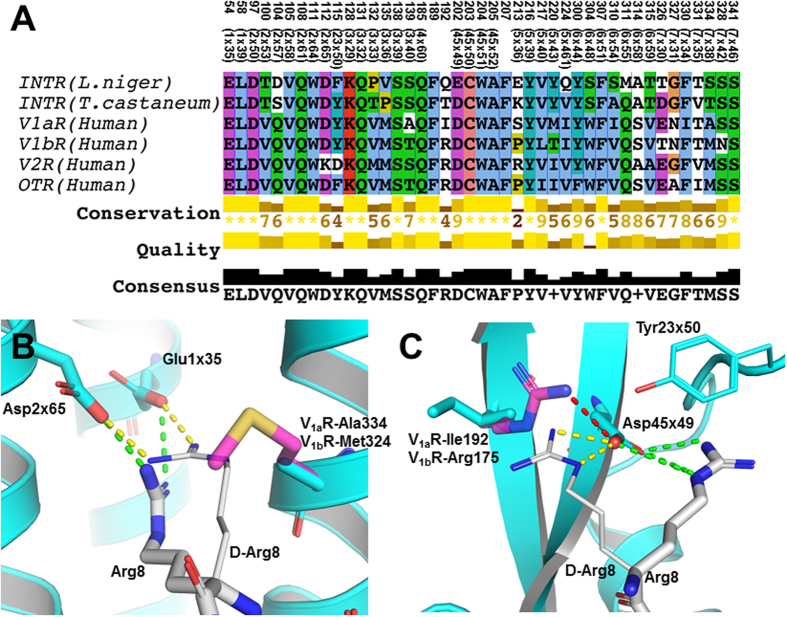
The selectivity of [D-Arg8]-inotocin for V_1a_R is conferred by a unique binding mode. (**A**) Sequence alignment of the binding site residues of the six investigated receptors, inotocin receptors *L. niger* and *T. castaneum*, as well as human V_1a_R, V_1b_R, V_2_R and OTR as defined by the receptor homology models. Together with the consensus sequence, the conservation score from 1–10 by physico-chemical properties^72^ and “*” indicating complete identity is given below the alignment. The sequence-based residue numbers are according to the V_1a_R, while the numbers in parenthesis are generic residue numbers from the GPCR database, GPCRdb.org^33^. (**B** and **C**) Models of the inotocin/[D-Arg8]-inotocin Arg8/D-Arg8 salt bridge interactions with the receptor residues Glu1 ×35 and Asp45×49, respectively. V_1a_R is represented as cyan cartoon and sticks, Arg8 and D-Arg8 are represented as thick and thin white sticks, respectively, and residues from V_1b_R are shown as magenta sticks. Putative salt-bridge interactions are indicated by the dashed lines; green between Arg8 and V_1a_R, yellow between D-Arg8 and V_1a_R and red to Arg 175 in V_1b_R^37^.

Following the characterization of [D-Arg8]-inotocin as a subtype selective vasopressin V_1a_-receptor ligand, we wanted to rationalize this effect in terms of key ligand interactions in the context of the receptor binding site. Analysis of sequence differences between the human vasopressin and ant/beetle inotocin receptors should provide insights into the observed differences in functional and binding selectivity. To pinpoint the amino acid positions in the receptors that are accessible by ligands, i.e. residues in the upper part of the transmembrane (TM) helices and the extracellular loop (ECL) 2 facing the common GPCR binding cavity, we constructed homology models of the six receptors (human OTR, V_1a_R, V_1b_R and V_2_R; *L. niger* and *T. castaneum* inotocin receptors) using the crystal structures of the μ-opioid and orexin receptors^31^,^32^ as templates. Based on these models, 43 positions with side-chains facing the spacious inter-helical cavity were extracted from the global sequence alignment to construct a local structure-based binding site alignment (Fig. 4A and Supplementary Fig. 4). This approach offers a way to identify both conserved and different sequence positions, which are likely to influence peptide binding, selectivity and function. The sequence similarity of the binding site residues of the three human vasopressin receptors is within 74–88%, between the ant and beetle inotocin receptor 77%, and between the vasopressin V_1a_/V_1b_ receptors and the two inotocin receptors 63%. In total, 21 (49%) positions of the binding cavity are conserved residues with similar properties in all receptors, e.g. Glu1×35, Lys3×29, Glu/Asp45×49 (GPCRdb generic numbering^33^), which explains the binding of native inotocin to 5 out of 6 receptors (except human V_2_R, where allosteric agonism was observed). Furthermore, the binding site alignment shows 6 positions in the V_1a_R, i.e. Ala139^(3×40)^, Ile192, Ser213^(5×36)^, Met220^(5×43)^, Ile330^(7×34)^ and Ala334^(7×38)^, which distinguish V_1a_R from V_1b_R and the inotocin receptors (Fig. 4A) and which could be responsible for the observed inactivity of native inotocin at V_1a_R while being a full agonist at V_1b_R (Table 2). Additionally, the 4^th^ position in ECL2 (Ile192 in V_1a_R) and position 7×38 contain an Arg and Met, respectively, in V_1b_R, V_2_R and OTR, while the corresponding residues in V_1a_R and the inotocin receptors have neutral and short side chains. This could be correlated to the lack of binding for [D-Arg8]-inotocin at the V_1b_R, V_2_R and OTR.

### Human serum stability of [D-Arg8]-inotocin

In addition to the valuable selectivity profile at the human oxytocin/vasopressin receptors, [D-Arg8]-inotocin was anticipated to be significantly more stable than its lead ligand inotocin as well as vasopressin due to dismantling the recognized trypsin Pro-Arg cleavage site by introducing the non-natural D-Arg residue^29^,^34^. The increased stability of [D-Arg8]-inotocin was confirmed in human serum by reversed-phase high performance liquid chromatography (RP-HPLC) in a 24 h time-course experiment (Supplementary Fig. 5). Native inotocin, vasopressin and melittin were used as controls. Whilst vasopressin (t_½_ = 1.0 h), inotocin (t_½_ = 2.8 h) and melittin (t_½_ = 0.7 h) were fully degraded within 24 h, > 70% of [D-Arg8]-inotocin was still present after 24 h.

### Concentration-dependent inhibition of human myometrial contractions by [D-Arg8]-inotocin

**Figure 5:**
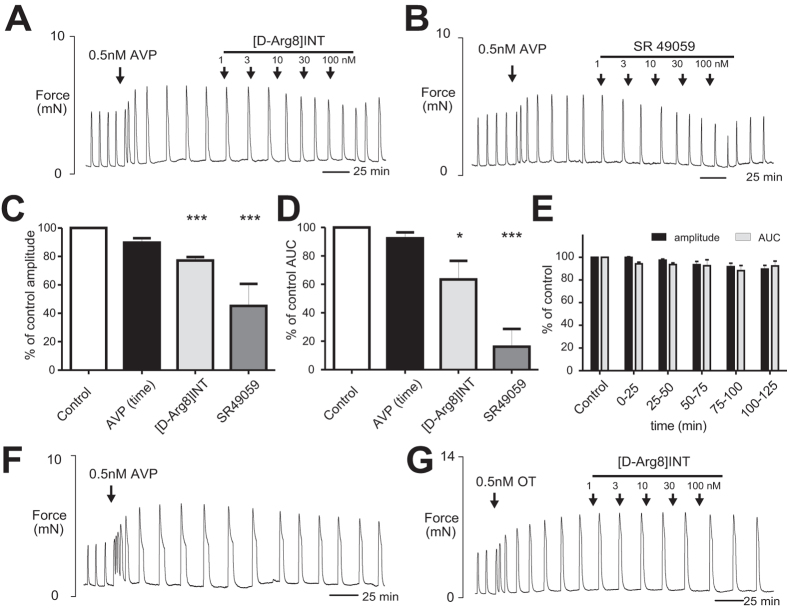
Uterine contractility inhibitory effects of [D-Arg8]-inotocin. Inhibitory effect of increasing concentrations of (**A**) [D-Arg8]-inotocin ([D-Arg8]INT) and (**B**) the commercially-available V_1a_R antagonist SR49059 on vasopressin-augmented (0.5 nM) myometrial contractions. Contraction amplitude and area-under-the-curve (AUC) under [D-Arg8]-inotocin treatment (100 nM) were significantly reduced (*P* < 0.05 and *P* < 0.001, respectively) as compared to control activity. As expected, SR49059 reduced both amplitude of contraction and AUC significantly at 100 nM (*P* < 0.001). The mean amplitude of contraction and AUC calculated for 100 nM [D-Arg8]-inotocin, SR 49059 or vasopressin (time-equivalent), expressed as a percentage of control where control equals 100% are shown in C and D. Application of 100 nM [D-Arg8]-inotocin significantly reduced contraction amplitude by 22.9 (±2.5%, *P* < 0.001) and AUC by 36.6 (±13.1%, *P* < 0.05). SR49059 (100 nM) significantly reduced contraction amplitude and AUC by 54.8 (±15.5%, *P* < 0.001) and 72.1 (±12.5%, *P* < 0.001), respectively. Contractions augmented by vasopressin (AVP) persisted without significant loss of amplitude or AUC for over 2 h during the time equivalent of experimental manoeuvers (**C**–**F**) and [D-Arg8]-inotocin has no inhibitory effect on contractions augmented with oxytocin (**G**). *P < 0.05, **P < 0.01, ***P < 0.001 (n = 5, one-way ANOVA, Tukey’s post hoc analysis).

To assess the functional relevance of this novel human V_1a_R antagonist we tested [D-Arg8]-inotocin in a well-recognised *ex vivo* human uterine contractility model^14^,^35^. After obtaining stable contractions of prepared tissue, human myometrium was stimulated with vasopressin (0.5 nM). The effects of [D-Arg8]-inotocin on these contractions were then investigated and compared to the small molecule V_1a_R antagonist SR49059 (relcovaptan)^10^. Both ligands caused a concentration-dependent decrease in vasopressin-augmented myometrial contraction amplitude and area-under-the-curve (Fig. 5A,B). For [D-Arg8]-inotocin, this became significant at 10 nM for amplitude (*P* < 0.01) (Supplementary Fig. 6A) and 100 nM for area-under-the-curve (*P* < 0.05) (Fig. 5D; Supplementary Fig. 6B). For SR49059, at 30 nM we observed a significant reduction in uterine contraction for both amplitude of contraction and area-under-the-curve (*P* < 0.05 and *P* < 0.001, respectively) (Supplementary Fig. 6C,D). Some contraction recovery was noted for both ligands upon washout (Fig. 5A,B). The effect of 100 nM [D-Arg8]-inotocin and SR49059 on amplitude of contraction and area-under-the-curve compared to the vasopressin (time-equivalent) and control activity are shown (Fig. 5C,D). Effects of [D-Arg8]-inotocin and SR49059 at other concentrations (1, 3, 10 and 30 nM) are presented in Supplementary Fig. 6. [D-Arg8]-inotocin significantly reduced contraction amplitude by 22.9% (Fig. 5C) and showed a clear reduction of area-under-the-curve by 36.6% (Fig. 5D). SR49059 (100 nM) significantly reduced contraction amplitude and area-under-the-curve by 54.8% (Fig. 5C) and 72.1%, respectively (Fig. 5D). Contractions under vasopressin alone however persisted without significant loss of amplitude or area-under-the-curve for the duration of the experimental protocol (Fig. 5C–F). [D-Arg8]-inotocin did not have any effect on myometrial contractions that were augmented with oxytocin (0.5 nM) (Fig. 5G).

## Discussion

The oxytocin/vasopressin GPCR family of receptors and neuropeptide ligands are part of a 600-million-year-old signalling system regulating many fundamental functions^36^. Advances in sequencing, molecular cloning and the expression of receptors in several heterologous systems have contributed to a renewed interest in structural and functional studies of members of this receptor family. The physiological complexity and therapeutic relevance of the oxytocin and vasopressin receptors render this receptor family as one of the most studied GPCRs. Therefore, oxytocin/vasopressin receptors represent interesting experimental models to study GPCR function and ligand development^9^. Linking the highly homologous receptor subtypes to their specific physiological and pathological functions has been a challenging and tedious process, particularly due to the lack of a complete set of receptor subtype-selective ligands^9^. To address this issue, we have recently proposed a strategy that focusses on the discovery and characterization of oxytocin/vasopressin-like ligands from nature and that takes advantage of the ancient character, high homology, conservation and distribution of the oxytocin/vasopressin signalling system throughout the animal kingdom, including humans^9^. Adopting this strategy has led to the discovery and characterization of an ant inotocin ligand pair in this work, which also formed an ideal template for the development of the novel, selective and stable human vasopressin V_1a_ receptor antagonist [D-Arg8]-inotocin. [D-Arg8]-inotocin is not only an excellent new research tool as will be discussed in the following paragraphs, but also may serve as a promising new starting point for future drug design and development efforts targeting V_1a_R-related disorders.

Previous mutagenesis studies on the V_1a_R in combination with vasopressin and its analogues have shown that residue 8 of vasopressin contributes to high affinity peptide binding in the V_1a_R^25^,^37^. In the present study, we utilized pharmacological measurements of the ant vasopressin-orthologue inotocin, its [D-Arg8]-inotocin analogue, and endogenous human vasopressin in heterologous systems expressing the human OTR, V_1a_R, V_1b_R and V_2_R, as well as two evolutionary-related inotocin GPCRs from ants and beetles. In a comparative approach, cell-based *in vitro* data were applied to receptor homology models to explain the observed pharmacological effects.

The engineered peptide ligand [D-Arg8]-inotocin is a potent, stable and competitive V_1a_R-antagonist with a 3,000-fold binding selectivity for the human V_1a_R over the other three subtypes, OTR, V_1b_R and V_2_R. We note that the observed partial activation of the human V_2_R by inotocin and [D-Arg8]-inotocin is likely due to direct allosteric agonism, which has been described for other GPCRs previously^38^,^39^,^40^,^41^. The insect-derived peptide probe [D-Arg8]-inotocin significantly reduced the contraction amplitude and area-under-the-curve in vasopressin-augmented tissue, albeit there were no detectable effects on myometrial contractions that were augmented with oxytocin. This effect is in line with the reduction in amplitude of contraction and area-under-the-curve with the commercially available small molecule V_1a_R antagonist SR49059 (relcovaptan)^10^, although [D-Arg8]-inotocin is less potent. From a therapeutic viewpoint, the inhibition of human V_1a_R and the partial activation of V_2_R receptor isoforms by [D-Arg8]-inotocin, although to date there is no compelling evidence for the expression of V_2_R receptor subtypes in human uterine tissue, could have synergistic effects leading to the reduction in contractility and supporting tocolysis^42^,^43^.

To provide a molecular explanation for the interesting pharmacological profile of [D-Arg8]-inotocin, we built homology models for the human OT, V_1a_, V_1b_ and V_2_ receptors as well as the two evolutionary-related insect receptors from ant and beetle to identify the binding site residue positions. To deduce structural requirements for the binding of the two nonapeptides inotocin and [D-Arg8]-inotocin, we reasonably assumed – supported by radioligand displacement data – that the conserved part of the peptide would engage and occupy the same space of the orthosteric receptor binding pocket of the receptors allowing us to obtain novel insights of the molecular role of the L- to D-Arg substitution. Our data clearly indicates that the stereochemistry of Arg8 is essential for binding to the proposed binding pocket of human OTR, V_1a_R and V_1b_R, while [D-Arg8]-inotocin only binds to the human V_1a_R and the two inotocin receptors without detectable affinity to the human V_1b_R, V_2_R and OTR. Interpretation of the observed pharmacological effects leads to the two hypotheses that either (i) the Arg8/D-Arg8 side chain interacts with different residues, with the putative interaction partner of Arg8 being conserved in all subtypes, while that of D-Arg8 only being present in the inotocin receptors and V_1a_R, or that (ii) the Arg8/D-Arg8 side chain interacts with the same position and that a nearby residue(s) hinders the interaction of the D-Arg8 side chain to the position of importance in three of the human GPCRs. Importantly, the observed effects of the Arg8 stereochemistry in inotocin/[D-Arg8]-inotocin does not apply to vasopressin/[D-Arg8]-vasopressin, as the first pair shows similar binding affinities in the V_1a_R (11 and 1.3 nM, Table 1), while previous studies have shown ~200-fold difference in affinity of the latter peptide pair (4.2 and 781 nM)^32^,^43^. While this does not exclude similar binding modes for inotocin and vasopressin, it clearly shows different binding modes for [D-Arg8]-inotocin and [D-Arg8]-vasopressin and different spatial locations of Arg8 in the binding site. Furthermore, neither vasopressin (Table 2), nor the non-selective vasopressin V_2_R agonist, desmopressin (1-desamino-8-D-arginine-vasopressin; dDAVP), activates inotocin receptors (Supplementary Fig. 7).

The binding site alignment does not pinpoint a unique position with an acidic or hydrogen bond-accepting residue in the binding site of [D-Arg8]-inotocin binding receptors, thereby weakening hypothesis (i) of different interaction partners for the guanidine moiety of Arg8/D-Arg8. Instead, the alignment revealed two positions, 7×38 and the 4^th^ residue in ECL2 (Ile192 in V_1a_R), which distinguish the [D-Arg8]-inotocin binding receptors from the non-binding ones. Importantly, these two positions are in proximity of the negatively charged Glu1×35 and Asp/Glu45×49, respectively, which are conserved within the vasopressin and inotocin receptors, and potential interaction partners to the side-chain of Arg8/D-Arg8. Residue Glu1×35 is located in the second helical turn and Asp/Glu45×49 in ECL2 next to Cys45×50 forming a disulphide bridge to TM3 and hence both positions are conserved in structure as well as in sequence. Of note, position 45×49 and 45×50 in ECL2 have been considered to be essential for high-affinity agonist binding and receptor activation and to be highly conserved throughout the neurohypophyseal hormone receptor subfamily of GPCRs^44^.

Compared to V_1b_R, V_2_R and OTR, where position 7×38 is a methionine, the short side chain residues Ala, Ser and Thr occupy this position in V_1a_R and the two inotocin receptors, respectively. The steric hindrance of the longer methionine side chain in combination with the stereochemistry of Arg8/D-Arg8 offers a potential explanation for the lack of [D-Arg8]-inotocin binding to V_1b_R, V_2_R and OTR, if Glu1×35 is a key interaction partner (Fig. 4B). Arg8 and D-Arg8 could approach the putative salt bridge partner from slightly different directions and, while the short side chains allow unhindered access of both, Met7×38 in V_1b_R, V_2_R and OTR may sterically interfere with D-Arg8 only. Additionally, the structural neighbour, Lys2×65 in V_2_R (Asp in the other receptors), provides a structural explanation for the marked reduction in affinity of inotocin in V_2_R compared to the other receptors (Table 1) likely due to a charge repulsion – an explanation that could also apply if the positively charged peptide C-terminus was the interaction partner of Glu1×35. Ile192 in V_1a_R is a structural neighbour to Asp/Glu45×49 on the adjacent β-strand and along with Gln and Thr at the two inotocin receptors, is a non-positively charged residue. In comparison, this same position holds an arginine in V_1b_R, V_2_R and OTR. Arginine in this position is likely to form a salt bridge to Asp45×49 in V_1b_R, V_2_R and OTR, blocking part of the interaction area of the aspartate (Fig. 4C). If the stereochemistry of Arg8/D-Arg8 results in a different location of the guanidine moiety relative to the Asp45×49, it is likely that the presence of an arginine in this position may prevent only [D-Arg8]-inotocin from forming a salt bridge, thus abolishing or severely impeding binding of this peptide in V_1b_R, V_2_R and OTR. This hypothesis also puts the Arg8 guanidine of inotocin in the vicinity of the structurally neighboring Tyr115^(23×50)^ in V_1a_R (Fig. 4C), which together with position 45×49 has also been hypothesized to interact with the Arg8 side chain of vasopressin^37^,^44^. Since both DAVP and dDAVP bind potently to V_2_R^37^, but [D-Arg8]-inotocin does not bind (Table 1), the observed affinity differences in V_1a_R, indicates overlapping binding modes of inotocin and vasopressin, but not of [D-Arg8]-inotocin and [D-Arg8]-vasopressin.

At a more general level, the function of a peptide on a receptor is not as logically reflected in the ligand-receptor interactions as the binding affinity, whereas our structural sequence alignment of the receptor binding site may indicate which residues are responsible for the pharmacological differences observed from the experimental data. Inotocin display ~20-fold higher potency at *L. niger* vs. the *T. castaneum* inotocin receptor inspite of similar binding affinities (Tables 1, [Table t2] and 2, [Table t2]). It is tempting to ascribe this observation to the unique glutamate in position 45×49 of the *L. niger* inotocin receptor, which may in general favour activation relative to the aspartate of the other receptors and, additionally, activation by the guanidine of inotocin relative to that of [D-Arg8]-inotocin. Moreover, this would further support position 45×49 as the crucial interaction partner of the Arg8/D-Arg8 side chain and not the alternative Glu1×35, which is conserved in all six receptors.

**Figure 6:**
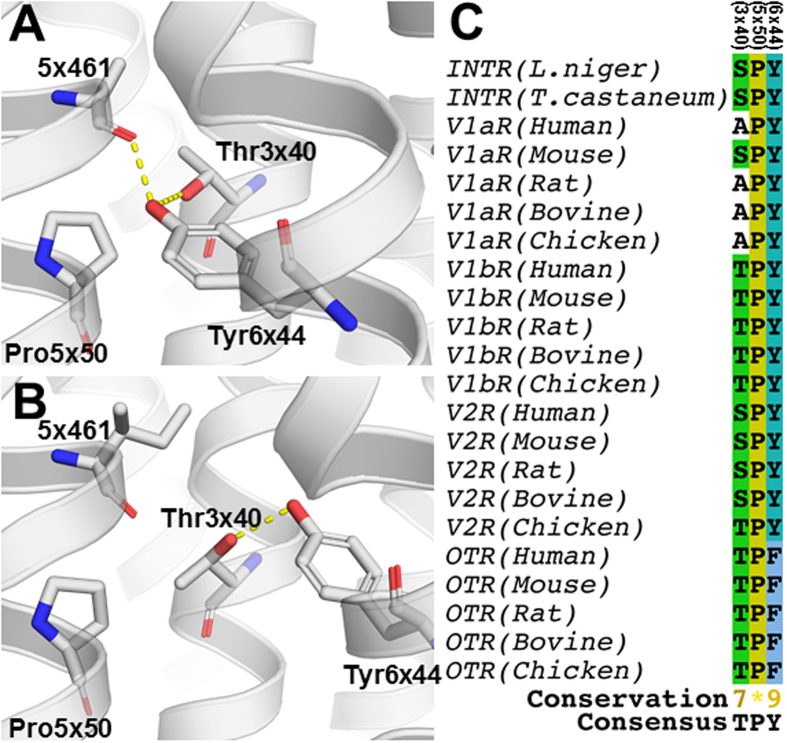
Putative structure and sequence homology of the conserved core triad. (**A**) Putative hydrogen bonding network (yellow dotted lines) of residues in position 3×40, 5×50 and 6×44 of V_1b_R (white cartoon and sticks) in an active receptor state by *in silico* amino acid exchange into the active state μ-opioid receptor crystal structure (PDB code: 5CM1)^45^. The hydrogen bond between Tyr6×44 and the backbone carbonyl of position 5×461 is accomplished by a large movement of TM6 relative to the inactive state. (**B**) Hydrogen bond between Thr3×40 and Tyr6×44 in the inactive state V_1b_R homology model. (**C**) Sequence conservation of positions 3×40, 5×50 and 6×44 in the studied receptors and vasopressin/oxytocin receptors from selected species. Interestingly, most V_1a_Rs lack the hydrogen bonding ability in position 3×40 as do the OTRs in position 6×44, which according to our model may result in different activation kinetics and thus different pharmacology. The consensus sequence, the conservation score from 1–10 by physico-chemical properties^72^ and “*” indicating complete identity is given below the alignment.

Another and more clear-cut pharmacological observation is that, in contrast to the agonistic effects of inotocin and [D-Arg8]-inotocin on the inotocin receptors (plus inotocin on V_1b_R), both peptides exert antagonist effects on V_1a_R. The search for sequence differences, which may offer an explanation for this observation, results in only two positions, 3×40 and 7×34, that clearly distinguish V_1a_R from V_1b_R and the two inotocin receptors. This lack of activation is clearly ligand dependent, as the related neuropeptide, vasopressin, potently activates V_1a_R indicating that the responsible amino acids are one or more of Tyr2, Phe3 and Gln4 in vasopressin, which differ from the corresponding positions in inotocin, Leu2, Ile3 and Thr4. Assuming that vasopressin, relative to inotocin, forms some additional contacts to V_1a_R that are responsible for activation, neither Ala139^(3×40)^ nor Ile330^(7×34)^ provides an explanation for this fact. However, position 3×40 in the narrow bottom part of the binding site has together with 5×50 and 6×44 been shown by the research team of Kobilka^45^ to form a ‘conserved core triad’ in other class A receptors. These three positions are dominantly Ile/Leu/Val, Pro and Phe/Tyr, respectively, and rearrange their hydrophobic packing during movement of TM6 and receptor activation. Additionally, the combination of NMR and mutagenesis experiments showed that increasing the van der Waals contact area by Val-to-Ile exchange in position 3×40 of a thermostabilised β_1_ adrenoceptor shifts the receptor towards an active state^46^. Considering a similar activation switch in the receptors studied here, we performed *in silico* exchange of positions 3×40 and 6×44 in the crystal structure of the active state μ-opioid receptor^45^ with those of V_1b_R (Fig. 6A). In our V_1b_R homology model of the inactive state, Tyr290^(6×44)^ and Thr122^(3×40)^ forms an inter-helical hydrogen bond (Fig. 6B). However, the movement of TM6 in the active state allows Tyr290^(6×44)^ to form a hydrogen bond to the backbone carbonyl of the residue in position 5×461. This position is a so-called helical ‘bulge’ residue, i.e. an additional 5^th^ residue in a helical turn^33^ and, together with the conserved Pro5×50, this changes the normal helical hydrogen bonding pattern making the backbone carbonyl accessible to Tyr290^(6×44)^ (Fig. 6A). Assuming a conformational change analogous to those observed in the crystal structures^45^ the conformation of Tyr290^(6×44)^ is likely further stabilized by an additional hydrogen bond to Thr122^(3×40)^. Among the receptors studied here, only V_1a_R lacks the hydrogen bonding residue in position 3×40 that potentially stabilizes an active receptor state (Fig. 6C). Thus, our data indicates that the lack of this hydrogen bond due to Ala139^(3×40)^ in V_1a_R results in an increased energy barrier of activation, which can explain the different pharmacological actions of inotocin and [D-Arg8]-inotocin on V_1a_R vs. V_1b_R and the two inotocin receptors. Interestingly, the mouse V_1a_R does contain a serine in position 3×40, which may explain observed pharmacological differences relative to the human receptor^47^. Additionally, mammalian oxytocin receptors have a phenylalanine in position 6×44 (Fig. 6), which also prevents formation of the hydrogen bonding network: this also agrees with the observed lack of activation of the human OTR by inotocin, despite the peptide binding. This further implies that vasopressin may have evolved to compensate for the increased activation energy barrier, particularly through residues Tyr2, Phe3 and Gln4 of the peptide mediating key contacts. This may be a fine example of co-evolution of peptide and receptor, i.e. arginine-vasopressin and the human V_1a_R.

More than 60 years have passed since the discovery, synthesis and characterization of oxytocin and vasopressin, yet there still remains a shortage of agonists and antagonists with good selectivity for the four known receptor subtypes^48^. The development of antagonists for vasopressin receptors is challenging due to the large degree of structural homology within the four receptor subtypes and their inter-species differences. Currently, there are no ligands in clinical use with selective antagonist activity for the V_1a_R over the V_1b_R and V_2_R. For this reason, novel strategies for ligand discovery and design represent a powerful option and a promising opportunity to outpace and effectively tackle this problem. Vasopressin antagonists are valuable pharmacological tools for investigating the pharmacological functions of the nonapeptide vasopressin. Previous studies have demonstrated that the carboxy-terminal position in vasopressin is a critical structural requirement for receptor activation but not binding^49^. To guide the rational design of novel peptide antagonists based on the moiety of the native ligand, it is necessary to identify conserved and non-conserved functional receptor domains involved in receptor binding and activation, such as residue positions 192 in V_1a_R, 3×40, 45×49, 6×44 and 7×38 that were described and analyzed in this study.

## Conclusion

Oxytocin/vasopressin receptors are promising targets for important diseases, including cardiovascular and neurological disorders. Despite extensive efforts there is demand for oxytocin/vasopressin ligands as therapeutics or modulators to probe the biological function of individual receptor subtypes. In a novel strategy that is broadly applicable to the development of selective ligands for the oxytocin/vasopressin family, we explored the evolutionary conservation of this signalling system to discover and subsequently engineer a competitive, subtype-selective and stable human V_1a_R-antagonist. We gained deeper insights into ligand-receptor pharmacology through integrated and iterative structure modelling and pharmacological evaluation. In fact, understanding the molecular mechanisms responsible for agonist and antagonist binding is critical and the information derived from our study may be useful for the rational design of potential therapeutic agents targeting oxytocin/vasopressin receptors. Furthermore, we have demonstrated the utility of the engineered inotocin ligand in a translational *ex vivo* human myometrial contractility study. Therefore, we consider that [D-Arg8]-inotocin has great potential to advance our understanding of V_1a_-receptor physiology and its associated role in disease. Overall, this study establishes proof-of-concept for exploiting insect-derived neuropeptide probes for human ligand development, which may be applied to other GPCRs.

## Methods

### Transcriptome analysis

For the transcriptome analysis, 96 *Lasius niger* workers were used from laboratory colonies established at the IST Austria, and reared under a 14 h day (27 °C) – 10 h night (21 °C) cycle. Total RNA was extracted from 48 pools of two workers each, using the Maxwell^®^ 16 Research instrument and the Maxwell^®^ 16 LEV simplyRNA Tissue Kit (Promega, Mannheim, Germany). After extraction, all 48 samples were pooled resulting in a single sample with an overall amount of 24 μg total RNA based on NanoDrop measurements (A.V. Grasse, unpubl.). The RNA was sent to Eurofins MWG GmbH (Ebersberg, Germany) for quality control, preparation of a 2x normalized, random-primed cDNA library and subsequent sequencing on the Illumina HiSeq 2000 platform, applying the 2 × 100 bp paired-end read module. The raw sequence reads were checked with FastQC (http://www.bioinformatics.babraham.ac.uk/projects/fastqc/, version 0.11.2), filtered and trimmed with PRINSEQ lite^50^ (version 0.20.4) with the following parameters: min_len 40, min_qual_mean 28, trim_tail_left 8, trim_tail_right 8, and trim_ns_right 1. To remove all rRNA, SortMeRNA^51^ (version 1.8) with the default eight databases and default parameters was applied. For *de-novo* transcriptome assembly of the high quality mRNA read pairs, Trinity^52^ (r2013-02-25) was used. The resulting contigs were tested for completeness via CEGMA^53^ (version 2.5). To identify contigs representing the precursor and receptors, a BLAST^54^ search via TBLASTN, in BLAST + (version 2.2.29) against the assembly as database was carried out. Default parameters with an e-value cut-off of 1e-4 were used. As query the amino acid sequences of the following inotocin precursors and receptors were used: *Harpegnathos saltator* (GenBank accession DAA35079.1, XM_011149198.1, XM_011153432.1), *Atta cephalotes* (DAA35080, XP_012061245.1), *Camponothus floridanus*^21^, *Acromyrmex echinatior* (XM_011067026.1, EGI60623.1), *Solenopsis invicta* (XM_011161311) and *Pogonomyrmex barbatus* (XM_011641193, XM_011641194.1, XM_011641195.1). The multiple alignments of the blast-hits with the query sequences were calculated with Clustalw^55^ and MAFFT^56^. For the phylogenetic reconstruction of described receptors versus the best blast hits, we aligned 30 well described receptors with the top blast hits based on e-value, with Clustalw. The phylogenetic tree was then calculated via maximum likelihood phylogenetic reconstruction with RAxML (version 8.2.4)^57^, with the PROTGAMMABLOSUM62 model and 100 bootstrap replicates. For re-rooting and visualization of the phylogenetic tree we used Dendroscope^58^.

### cDNA cloning of the inotocin precursor

The full length precursor was obtained via Rapid Amplification of 3′ cDNA Ends (3′ RACE) technology. RNA of whole workers from laboratory colonies was extracted using Quick-RNA^TM^ MiniPrep kit (Zymo Research, Irvine, USA) and reverse transcription was done using High-Capacity cDNA Reverse Transcription Kit (Applied Biosystems, Carlsbad, USA) and polyT primer 5′-GGCCACGCGTCGACTAGTACTTTTTTTTTTTTTTTTT-3′. First PCR was performed using Phusion Hot Start II polymerase (Thermofisher Scientific, Waltham, USA) and primers polyT-end CTACTACTACTAGGCCACGCGTCGACTAGTAC and FW ATGCTAAAGAAGCTTGTCATTTTTGCGAG. Nested PCR was carried out using the first PCR product as template with a FW-nested primer 5′-AATTTTCCTGAGTTACGCTTGTTTGATTAC-3′ and the polyT-end primer. PCR fragments (~500 bp) were extracted, purified using GeneJET Gel Extraction kit (Thermofisher Scientific, Waltham, USA) and sequenced at LGC Genomics (Berlin, Germany) using the FW-nested primer. After determining the unknown 3′ sequence, one more PCR was performed using FW and Seq-Rev AAGTTGAAACATCAAAGTAATAAGCAAAAACTATG primers and cDNA as template. The obtained PCR fragment (full length inotocin precursor) was directly sequenced using the Seq-Rev primer to obtain the full length reading frame of the precursor sequence.

### cDNA cloning of inotocin receptor

The cDNA of the ant inotocin receptor was obtained by reverse transcription using the OneStep RT-PCR kit (Qiagen, Venlo, The Netherlands) and RNA isolated from *L. niger* as a template; the following primers were used: 5′-ATGTCGTATGACTCGAATACGTC-3′ (sense) and 5′-TCAGCCGAATATCTTTGAACTCG-3′ (antisense). Blunt end ligation of the PCR product with pJET1.2 vector was carried out at 4 °C (Clone Jet PCR cloning kit, Thermo Scientific Waltham, USA). Successful cloning was determined by digest using restriction endonuclease BglII (Thermo Scientific, Waltham, USA) and DNA sequencing of the plasmid containing the amplification product corresponding to the full length receptor (Microsynth AG, Balgach, Switzerland). For cellular expression, the receptor cDNA was cloned into the pEGFP-N1 vector at the restriction sites EcoRI and KpnI using the primers 5′-ATTCGAGAATTCATGTCGTATGACTCGAATACG-3′ (sense) and 5′-ACGCACGGTACCGTGCCGAATATCTTTGAACTCGCAAGTCGTGAAATTCTAG-3′ (antisense). This resulted in a plasmid encoding the *L. niger* inotocin receptor together with a C-terminal GFP fusion protein. The cDNA pXN-INTR from *T. castaneum* was kindly provided by Yoonseong Park (Department of Entomology, Kansas State University^59^,^60^). Similarly, the beetle inotocin receptor was cloned into pEGFP-N1 for cellular expression utilizing the KpnI and AgeI restriction sites and the primers 5′-ATTGGAGGTACCATGGACATCTCTGAGAACTCTACG-3′ (sense) and 5′- ATCGTCACGACGACCACCCCACCGGTCGCCACCATGGTG-3′ (antisense). Human receptor constructs encoding for the OTR, V_1a_R, V_1b_R and V_2_R were prepared and used as reported previously^35^.

### Database deposition

Nucleotide sequences corresponding to the coding sequences of the *L. niger* inotocin prepro-hormone and receptor were deposited in GenBank under the accession numbers KX266837 and KX266836, respectively. The raw sequence read files were deposited under the bioproject PRJNA352917; the sequence reads can be found in the short read archive: accessions SRR5007208 and SRR5007267.

### Peptide synthesis

Peptides were assembled manually *via* Boc-SPPS using the HBTU-mediated *in situ* neutralization protocol^61^,^62^. After HF cleavage^63^, the crude peptides were purified by preparative RP-HPLC (Vydac C_18_ column, 300 Å, 10 μm, 250 × 21.2 mm) using a linear gradient of 0–50% B (solvent A, H_2_O/0.05% TFA; solvent B, 90% CH_3_CN/10% H_2_O/0.043% TFA) in 50 min at 8 mL min^−1^ while monitoring UV absorbance at 226 nm. Air oxidation was carried out in 0.1 M NH_4_HCO_3_ buffer at 25 °C and a peptide concentration of 100 μM. Oxidation, deprotection and degree of purification were monitored by mass spectrometry (MS) on a LCT-TOF mass spectrometer equipped with an electro-spray ionization source (ESI), by analytical RP-HPLC using a Vydac (Grace, Epping Vic, Australia) C_18_ column (300 Å, 5 μm, 250 × 4.6 mm) at 214 nm and by ESI-LC-MS on a Phenomenex (Torrence, USA) Jupiter LC-MS C_18_ column (90 Å, 5 μm, 250 × 2 mm) on a SCIEX QSTAR Pulsar QqTOF mass spectrometer (Applied Biosystems, Carlsbad, USA) equipped with an atmospheric pressure ionization source, running a linear gradient 0–50% B over 50 min with a flow rate of 1 mL min^−1^ for RP-HPLC and 200 μL min^−1^ for LC-MS. The synthesized peptides were of >95% purity determined by analytical RP-HPLC monitored at 214 nm.

### [^3^H] inotocin synthesis

Fmoc-L-[4,5-^3^H] leucine obtained by catalytic tritiation of Fmoc-4,5-dehydro-L-leucine (S.A. = 112.6 Ci mmol^−1^) was added to the protected precursor peptide (CLITNCPRG-NH_2_) on PAL resin (Bachem, Bubendorf, Switzerland) using a standardised Fmoc protocol^64^. After the addition of a terminal cysteine residue the peptide was cleaved from the resin, deprotected and the disulfide bond was formed by bubbling air through the 0.1 M buffer solution (AcONH_4_, pH = 8.4) of the peptide. After radio-HPLC purification the [^3^H] inotocin (C[4,5-^3^H]LITNCPGR-NH_2_, radiochemical purity >99.9%; specific activity = 55.7 Ci mmol^−1^) was prepared in a solution of H_2_O:EtOH 4:1 (v/v) at 1 mCi mL^−1^.

### Cell culture and transient receptor expression

Chinese Hamster Ovary cells (CHO) were cultured in Ham’s F12 media supplemented with 10% fetal bovine serum (FBS), 1% PenStrep (all from Sigma-Aldrich, Munich, Germany) and grown in a humidified atmosphere of 37 °C, 5% CO_2_ and passaged on average twice per week. CHO cells were used for expression of inotocin receptor (*L. niger, T. castaneum*). Cell transfections were carried out with 10 μg of plasmid using Lipofectamine LTX (Life Technologies, Invitrogen, Carlsbad, USA) for invertebrate receptors. For the expression of human OTR, V_1a_R, V_1b_R and V_2_R, HEK293 cells were transfected using jetPRIME (Polyplus Transfections, Illkirch-Graffenstaden, France)^35^. Cells were maintained in DMEM (GE Healthcare Hy Clone™, Logan, USA) supplemented with 10% FBS and 1% PenStrep. Fluorescence and receptor expression was confirmed via epifluorescence microscopy 24 h post-transfection.

### Radioligand binding assays

Binding experiments were performed on transient receptor expressing membrane preparations (10–50 μg per assay), which were incubated with radioligand and peptide in 100 μL buffer containing 9 mM MgCl_2_, 25 mM Hepes, 0.15 mM bacitracin, 0.0015% (w/v) apoprotinin and 0.1% (w/v) bovine serum albumin. [Tyrosyl-2,6-^3^H]oxytocin, 47.4 Ci mmol^−1^ and [[phenylalanyl-3,4,5-^3^H]vasopressin, 61.2 Ci mmol^−1^ were from PerkinElmer Life Sciences, Warwick, USA; synthesis of [^3^H] inotocin was described above. Displacement of radioactive ligand was assayed in the presence of logarithmically-spaced concentrations of competing ligand or control oxytocin/vasopressin peptides. After 2 h at 20 °C, the reaction was terminated by rapid filtration over glass fibre filters (102 × 258 mm) (Filtermat A Wallac) previously soaked for 5 min in polyethylenimine 0.3% (v/v) in TRIS buffer (25 mM), to minimize unspecific binding. Unbound ligands were washed off (Skatron Cell Harvester). Displacement of radioactive ligand was assayed in the presence of saturating concentrations (10 μM) of oxytocin, vasopressin or inotocin, respectively, to determine non-specific binding. For the displacement at the human OTR, V_1a_R, V_1b_R and V_2_R the normalization to 100% refers on average to 0.49, 1.4, 0.36, 1.1 pmoles of ligand bound per milligram of membrane for human OTR, V_1a_R, V_1b_R and V_2_R, respectively. Bindings of [^3^H] AVP and [^3^H] OT were performed with a radioligand concentration dependent on the *K*_d_ of each receptor subtype (1.5, 0.6, 0.1 and 1.2 nM for OTR, V_1a_R, V_1b_R and V_2_R). Membranes (30–50 μg) expressing human OTR, V_1a_R, V_1b_R and V_2_R, were assayed for displacement with an excess of inotocin or [D-Arg8]-inotocin, and vasopressin or oxytocin (10^−11^ to 10^−5^ M) as positive control for each receptor (n ≥ 3, except where otherwise stated). For the displacement at the insect receptors the normalization to 100% refers on average to 0.65 and 0.69 pmoles of ligand bound per milligram of membrane for inotocin receptor from *L. niger* and *T. castaneum*, respectively. Bindings of [^3^H] inotocin to membranes (10 μg) expressing INTR from *L. niger*, or [^3^H] inotocin to membranes (10 μg) expressing INTR from *T. castaneum*, respectively, were assayed for displacement with an excess of inotocin (10^−11^ to 10^−5^ M) (n ≥ 3, except where otherwise stated). *K*_d_ values of inotocin receptors were determined from saturation binding experiments to be 0.9 nM (*L. niger*) and 2.7 nM (*T. castaneum*). Specific binding was calculated by subtracting the non-specific binding from the total binding. *IC*_50_ values were obtained by fitting the data to a three-parameter logistic Hill equation. Inhibition constants (*K*_i_) were calculated from *IC*_50_ using the method by Cheng and Prusoff^65^.

### Functional receptor activation assays

Activation of G_q_-signalling via the human V_1a_R, V_1b_R and OTR, as well as the insect INTR was measured by inositol-1-phosphate (IP_1_) quantification using an IP_1_ Tb kit (Cisbio, Codolet, France). Transfected cells were seeded in 96 well-plates, with a cell density of 10^4^ cells per well. Cell culture media was removed from the wells after 6 h and 70 μL of peptide of interest, prepared in IP_1_ 1X Stimulation Buffer at the desired concentrations, was added. Samples were measured in technical triplicates. After 1 h incubation at 37 °C, 15 μL of IP_1_-d2 was added, following the same volume of Ab-cryptate. After 1 h of incubation, activation was GPCR quantified via fluorescence measurement (SynergieH4, Biotek Instruments, Winooski, USA) using the ratio 665/620 nm. Activation of G_s_-signalling via the human V_2_R was measured using a luciferase reporter assay. HEK293 transiently expressing V_2_R were co-transfected with the pGL4.29 [luc2P/CRE/Hygro] (Promega, Mannheim, Germany) plasmid. After ligand stimulation, 6 h incubation at 37 °C, medium was removed and cells were kept at −80 °C for at least 12 h before lysis. The measured luciferase counts (SynergieH4) were corrected using the fluorescent intensity per well and normalized to the maximum activation of oxytocin/vasopressin peptides. For cell-based functional assays (luciferase and IP_1_), subtraction of the negative control (stimulation buffer) was applied to all data set. The normalization was performed to the highest value of the positive control from at least 4 independent experiments. Data were fitted using GraphPad Prism 5.0 with an equation for non-linear regression (sigmoidal, slope fit of 1). Values are reported as mean ± SEM. For Schild regression analysis, each concentration-ratio (r) was calculated as the agonist *EC*_50_ in the presence of a respective concentration of [D-Arg8]-inotocin by the *EC*_50_ of the agonist alone. The logarithm of the concentration-ratio (r-1) has been plotted vs. the logarithm of the respective concentration of [D-Arg8]-inotocin to obtain the pA2 value, which is defined as the negative logarithm of the molar concentration of the antagonist producing a two-fold shift in the agonist concentration-response curve (r = 2).

### GPCR homology modelling and *in silico* analysis

Template selection for the homology modelling was performed in GPCRdb^66^ using the human V_1a_R sequence as the query and selecting the μ-opioid^31^ and orexin^32^ crystal structures downloaded from the Protein Data Bank (rcsb.org/pdb)^67^ as templates. Sequences of the four human receptors and the beetle receptor were downloaded from the UniProt Knowledgebase uniprot.org^68^ (V_1a_R: P37288, V_1b_R: P47901, V_2_R: P30518 and OTR: P30559; *T. castaneum* D6WPA3), and the ant inotocin receptors was obtained by transcriptome mining and confirmed by PCR and sequencing. Multiple sequence alignment between V_1a_R, V_1b_R, V_2_R, OTR and the two invertebrate templates was created by manually adjusting the structure based sequence alignment from GPCRdb and adding the two inotocin receptors using ClustalX^55^. Homology models were constructed in MODELLER^69^, version 9.15; since they are only used for comparing structural positions, the optimization was disabled by using the “very_fast” keyword. The residues of the inter-helical binding site was defined in PyMOL (The PyMOL Molecular Graphics System, Version 1.8 Schrödinger, LLC.) by the residues comprising the van der Waal surface within the helical bundle and the surface of the ECL2 nearest to the cavity, thus extracted from the overall sequence alignment. The inotocin and [D-Arg8]-inotocin fragments used to illustrate the selectivity hypotheses were built and manually inserted into the binding site in Maestro (Maestro, version 10.3, Schrödinger, LLC, New York, NY, 2015). *In silico* amino acid exchange was performed using the mutagenesis wizard in PyMOL on the μ-opioid active state crystal structure^45^ downloaded from the PDB.

### Serum stability assays

Serum stability was tested as described previously^70^. Briefly, serum was obtained prepared from human blood (self-donor), allowed to coagulate for 2 h at 23 °C and centrifuged at 12,000 × g for 10 min. Peptides (100 μM) were incubated in serum for 2, 4, 6, 8, 10 and 24 h at 37 °C. To evaluate peptide stability, aliquots (25 μM) were analysed via RP-HPLC (UltiMate 3000, Thermo Scientific Dionex, Vienna, Austria) on a C_18_ column (Phenomenex Kinetex, Aschaffenburg, Germany) with a 3% gradient using 0.1% (v/v) TFA and 90/10/0.1% (v/v/v) acetonitrile/H_2_O/TFA as eluents. Half-life (t_1/2_) of peptides was calculated using non-linear regression analysis using Graph Pad Prism.

### Human myometrial contractility assays

Biopsies of human myometrium were obtained during pre-labour elective Caesarean Section (CS) delivery at term gestation (38–41 weeks) at Liverpool Women’s Hospital NHS Foundation Trust, Liverpool UK. At surgery, a biopsy measuring approx. 1 cm^3^ was cut from the upper lip of the lower uterine incision site and placed into Hanks Balanced Salt solution at 4 °C. All women provided written informed consent and ethical approval was sought and granted by the Local Research Ethics Committee (REC Ref: 10/H1002/49) and by the Research and Development Director of Liverpool Women’s NHS Foundation Trust, Liverpool, UK. Multiple strips were dissected and measured as previously described^35^,^71^. Data were analysed using OriginPro9.0 Software (OriginLab, Northampton, USA). Contractile activity was measured by calculation of the integral area-under-the-tension-curve (AUC, arbitrary units) and mean maximum amplitude of contraction (expressed in mN). Contractile activity in the final 25 min of vasopressin or oxytocin exposure, preceding the addition of the first concentration of [D-Arg8]-inotocin or SR49059 was taken as control activity. The activity under each concentration (or time period equivalent for vasopressin) were similarly calculated and expressed as a percentage of this control period (i.e. control activity is equal to 100%). Values represent the mean ± SEM where ‘n’ is the number of samples, each representing a different woman. All methods were performed in accordance with the relevant guidelines and regulations.

## Additional Information

**How to cite this article:** Di Giglio, M. G. *et al*. Development of a human vasopressin V_1a_-receptor antagonist from an evolutionary-related insect neuropeptide. *Sci. Rep.* **7**, 41002; doi: 10.1038/srep41002 (2017).

**Publisher's note:** Springer Nature remains neutral with regard to jurisdictional claims in published maps and institutional affiliations.

## Supplementary Material

Supplementary Information

## Figures and Tables

**Table 1:** Affinity (*K*_i_ in nM) of inotocin, [D-Arg8]-inotocin and vasopressin on insect and human receptors.

Peptide	Sequence	inotocin R (*L. niger*)	inotocin R (*T. castaneum)*	human V_1a_R	human V_1b_R	human V_2_R	human OTR
inotocin	CLITNCPRG*	6.0 ± 2.8	11 ± 3.9	11 ± 4.4	12 ± 6.5	>10^5^	62 ± 37
vasopressin	CYFQNCPRG*	>10^5^	>10^5^	2.6 ± 0.2	1.2 ± 0.6	4.2 ± 1.3	1.8 ± 0.5^†^
[D-Arg8]-inotocin	CLITNCPrG*	61 ± 10^#^	217 ± 77	1.3 ± 0.4	>10^5^	>10^5^	>4000

*C-terminal amide; ^†^value is for oxytocin at the oxytocin receptor; affinity (*K*_i_) data are indicated as mean ± SEM from at least 3 independent experiments (except ^#^n = 2); *K*_i_ values were calculated from *IC*_50_ values according to Cheng and Prusoff^65^, assuming *K*_d_ values of 0.6 nM for V_1a_R, 0.1 nM for V_1b_R, 1.2 nM for V_2_R and 1.5 nM for OTR. *K*_d_ values of inotocin receptors were determined from saturation binding experiments to be 0.9 nM (*L. niger*) and 2.7 nM (*T. castaneum*).

**Table 2 t2:** Potency (*EC*_50_ in nM) of inotocin, [D-Arg8]-inotocin and vasopressin on insect and human receptors.

Peptide	Sequence	inotocin R (*L. niger*)	inotocin R (*T. castaneum)*	human V_1a_R	human V_1b_R	human V_2_R	human OTR
inotocin	CLITNCPRG*	0.022 ± 0.006	0.35 ± 0.05	>10^5^	56 ± 9.7	>1500	>10^5^
vasopressin	CYFQNCPRG*	>10^5^	>10^5^	0.13 ± 0.02	0.6 ± 0.07	175 ± 76	41 ± 39
[D-Arg8]-inotocin	CLITNCPrG*	1.2 ± 0.7	12 ± 3.5	>10^5^	>10^5^	164 ± 26	>10^5^

*C-terminal amide; functional receptor activation (*EC*_50_) is indicated as mean ± SEM from at least 3 independent experiments.
