# Supplementary material for: Development of a human vasopressin V1a-receptor antagonist from an evolutionary-related insect neuropeptide
Source: Sci Rep. 2017 Feb 1;7:41002. doi: 10.1038/srep41002 (PMC5286520; doi:10.1038/srep41002)
Supplement: Supplementary Information [file srep41002-s1.pdf]

Supporting Information

Development of a human vasopressin V_{1a}-receptor antagonist from an evolutionary-related insect neuropeptide

Maria Giulia Di Giglio, Markus Muttenthaler, Kasper Harpsøe, Zita Liutkeviciute, Peter Keov, Thomas Eder, Thomas Rattei, Sarah Arrowsmith, Susan Wray, Ales Marek, Tomas Elbert, Paul F. Alewood, David E. Gloriam and Christian W. Gruber*

**Correspondence and requests for materials should be addressed to C.W.G. (email: christian.w.gruber@meduniwien.ac.at* or *c.gruber@uq.edu.au*)

Supplementary Figures

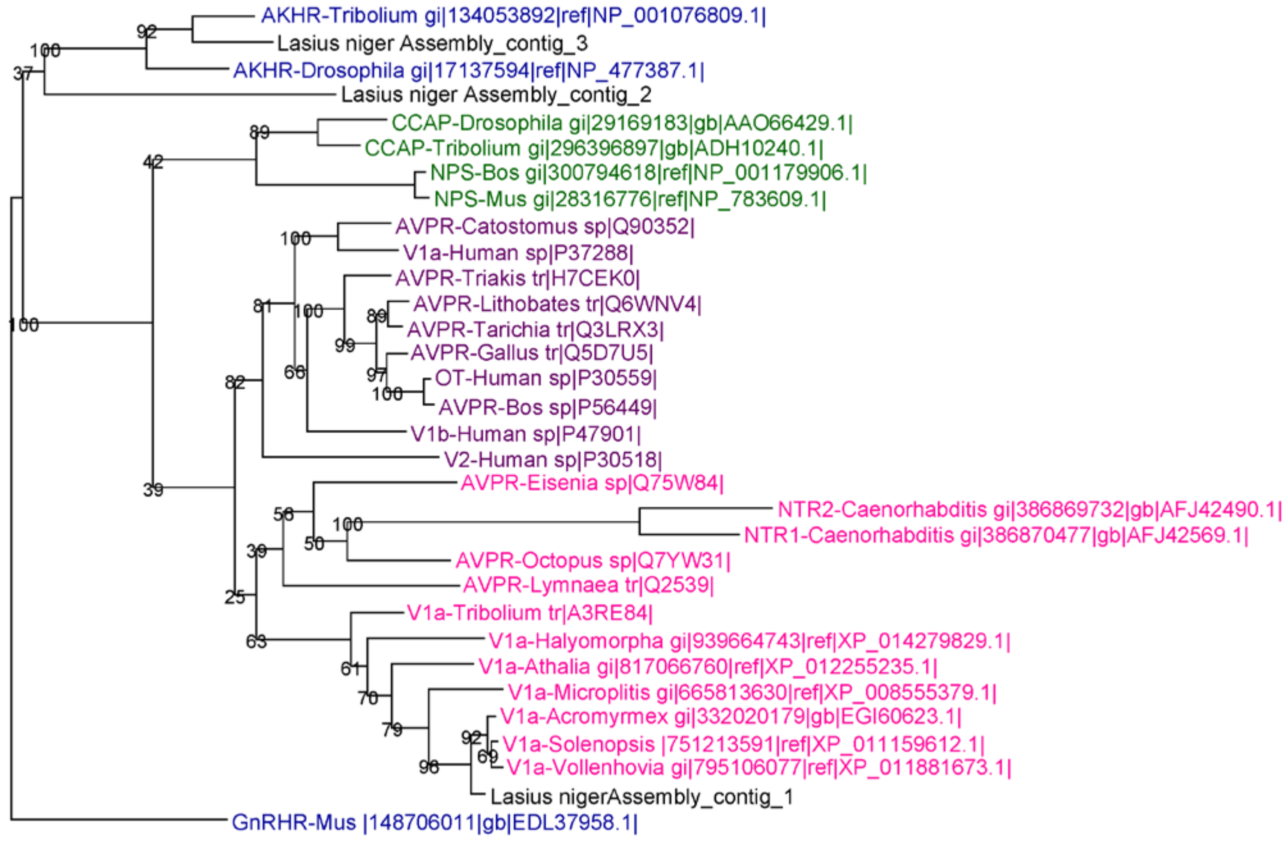

Supplementary Fig. 1. Phylogenetic tree of oxytocin/vasopressin (OT/AVP) receptors. Published invertebrate and vertebrate receptors were used together with the top three blast hits from the *L. niger* transcriptome (*Lasius niger* Assembly_contigs_1-3, shown in black) to reconstruct the phylogenetic relationship. Only the best blast hit of *L. niger* (*Lasius niger* Assembly_contig_1) clusters together with OT/AVP receptors in the same branch, while the other two hits cluster with invertebrate adipokinetic hormone receptors (AKHR). Vertebrate OT/AVP receptors are shown in violet and invertebrate OT/AVPR receptors are shown in pink. Putative OT/AVP-like ant receptors are shown in pink. Vertebrate neuropeptide S receptors (NPSR)/invertebrate crustacean cardioactive peptide receptors (CCAPR) are shown in green and vertebrate gonadotropin-releasing hormone receptors (GnRHR)/invertebrate AKHR are shown in blue. According to the study of Pitti and Manoj[1] the mouse gonadotropin-releasing hormone receptor (GnRHR) was used as outgroup. Numbers at nodes indicate confidence values and ExPASy/Genbank entry IDs of sequences are listed next to the receptor names.

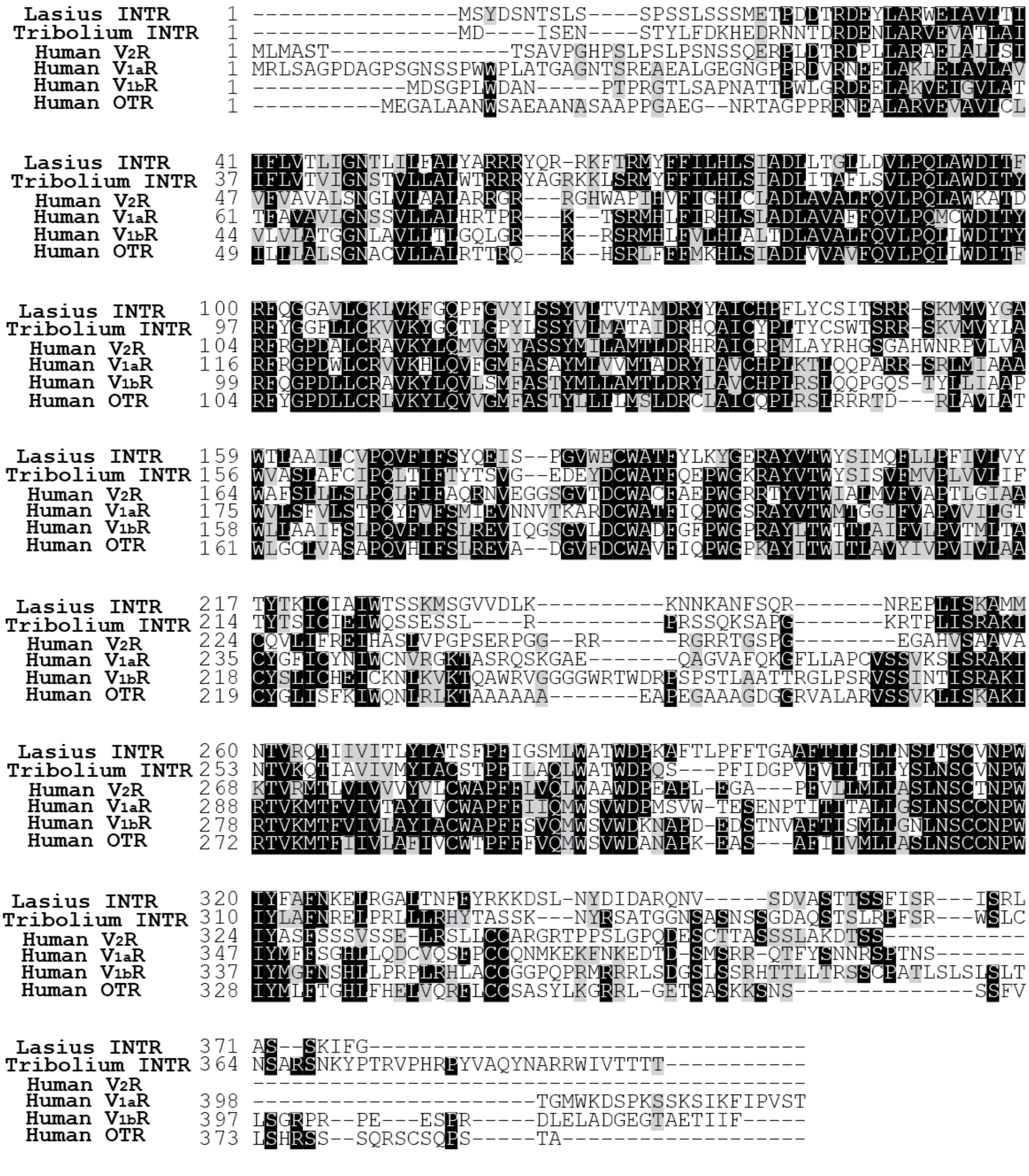

Supplementary Fig. 2. Multiple sequence alignments between inotocin receptors (*L. niger*, *T. castaneum*) and human V_2R, $V_{1a}R$, $V_{1b}R$ and OTR. FASTA sequences were aligned through Clustal Omega and shown in Boxshade format. Colour coding is defined as follows: residues that are similar but non-identical are highlighted in grey; identical residues are highlighted in black.

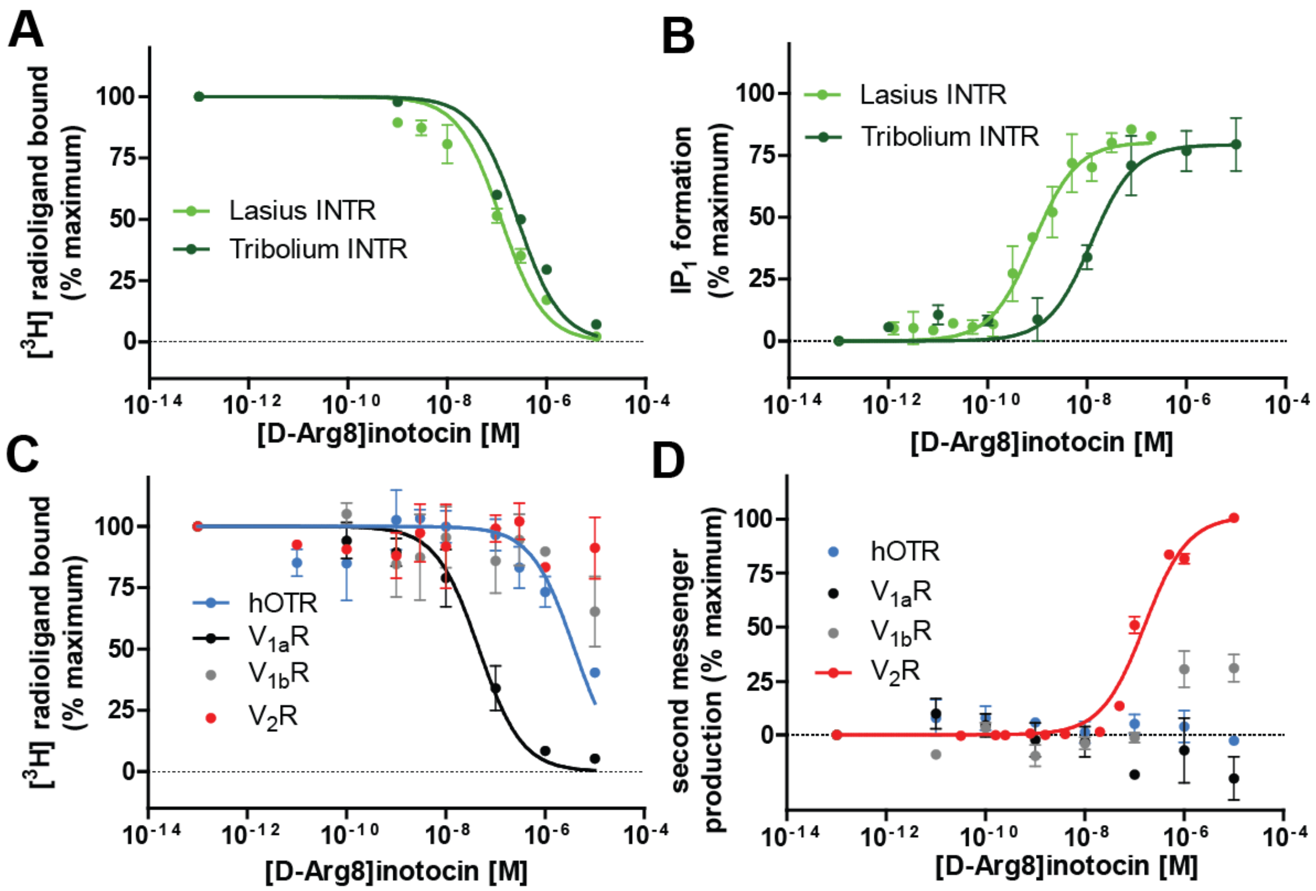

Supplementary Fig. 3. Receptor pharmacology of [D-Arg8]-inotocin at inotocin and human oxytocin/vasopressin receptors. (A) Concentration-dependent displacement binding curves of inotocin at inotocin receptors (INTR) from *Lasius niger* (●) (n = 2), *Tribolium castaneum* (●) (n = 3). (B) Concentration-response curves of [D-Arg8]-inotocin at INTR from *L. niger* (n = 4) and *T. castaneum* (n = 3) through quantitation of increased intracellular IP_1. (C) Concentration-dependent displacement binding curves of inotocin at human OTR (●), $V_{1a}R$ (●), $V_{1b}R$ (●) and V_2R (●) (n = 3). (D) Concentration-response curves of [D-Arg8]-inotocin at human OTR, $V_{1a}R$, $V_{1b}R$ and V_2R (n ≥ 3). Specific binding was calculated by subtraction of non-specific binding from total binding and normalized to the percentage (%) of maximal binding. Detailed descriptions of radioligand concentrations, membranes expressing receptors and dissociation constants are provided in the Methods section. Receptor activation was measured by IP_1 assays for the G_q-coupled receptors (human OTR, $V_{1a}R$, $V_{1b}R$) and luciferase reporter assay with specific CRE response element for the G_s-coupled human V_2R, as described in Methods. Each data point was normalized to percentage of maximal activation, detected at the highest endogenous ligand concentration, being inotocin for INTR, vasopressin for $V_{1a}R$, $V_{1b}R$, V_2R and oxytocin for OTR. Data is shown as mean ± SEM and fitted by nonlinear regression (sigmoidal, three-parameters, Hill slope of 1).

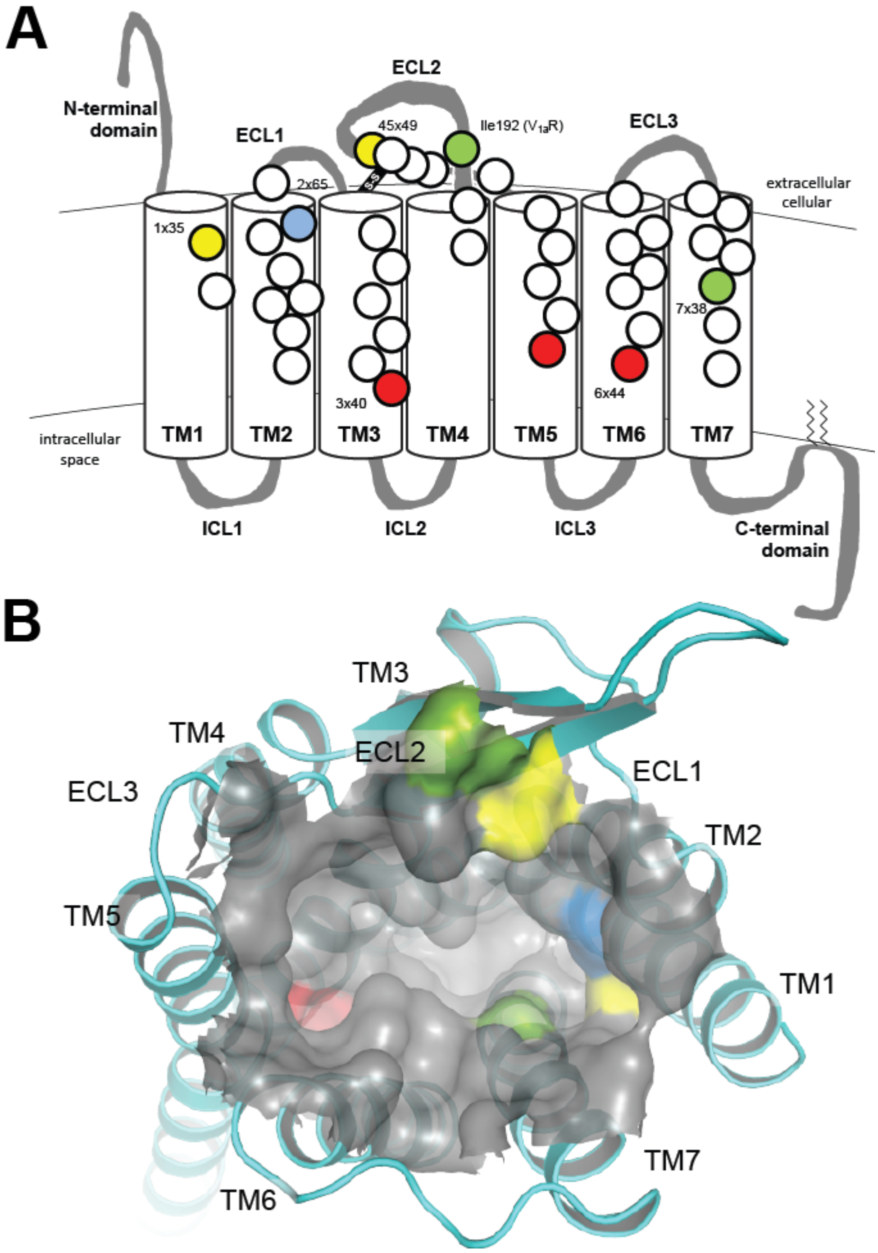

Supplementary Fig. 4. Structural representation of the human $V_{1a}R$ homology model binding site. (A) 2-dimensional cartoon representation of receptor structure showing relative positions of residues identified from sequence and structural alignments to comprise the predicted binding pocket (circles). Key residues predicted to discriminate the binding and function of inotocin and [D-Arg8]-inotocin are highlighted (coloured circles). Potential binding residues of Arg8/D-Arg8 are shown in yellow; proposed Lys in V_2R that impairs inotocin binding is shown in blue; proposed residues of the activation triad are shown in red; residues in human $V_{1b}R$, V_2R, and OTR that potentially impair D-Arg8 binding are presented in green. (B) Van der Waals surface (grey transparent surface) of the binding site residues highlighted in panel A) is presented in the $V_{1a}R$ homology model (cyan cartoon). One continuous cavity is observed within the upper part of the transmembrane domain comprised of 43 residue positions in TM1-7 and ECL1-2.

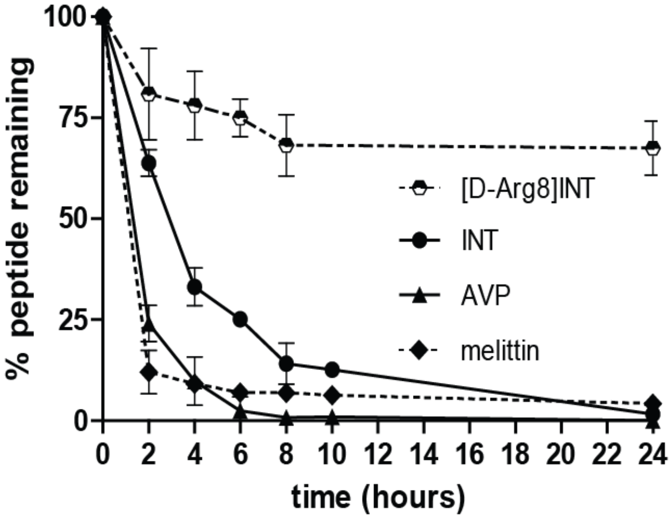

Supplementary Fig. 5. Human serum stability of the inotocin D-analogue. [D-Arg8]-inotocin ([D-Arg8]INT), inotocin (INT), vasopressin (AVP) and melittin (100 µM) were incubated in human serum and their stability monitored via HPLC over a time course of 2, 4, 6, 8, 10 and 24 h. Melittin, a haemolytic peptide from the bee venom was used as a positive control for peptide degradation. Area under the curves of samples was determined and correlated to the negative control analyte, which was dissolved in 0.1% TFA; thus they were not subject to degradation and were assumed therefore as 100%. Peptide stability was expressed in percentage of peptide remaining.

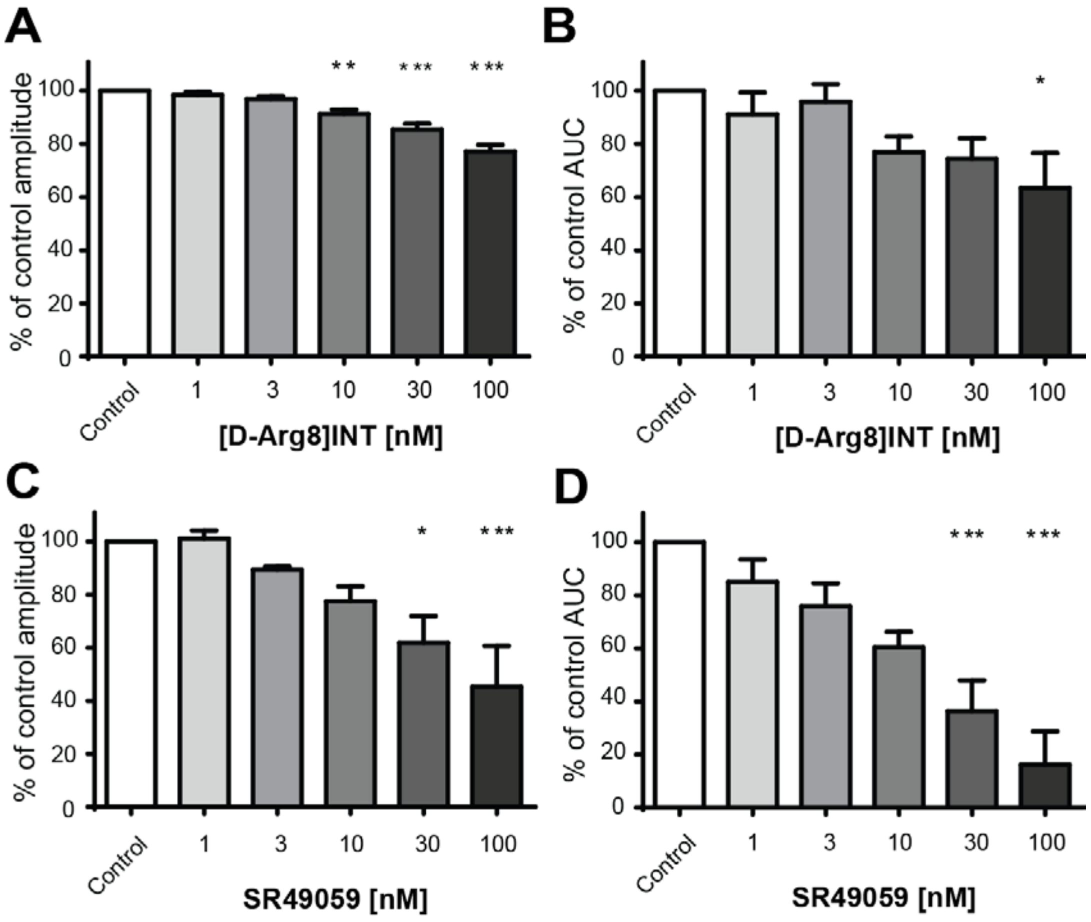

Supplementary Fig. 6. Concentration-dependent inhibitory effects of [D-Arg8]-inotocin and SR49059 on vasopressin-augmented (0.5 nM) *ex vivo* uterine contractions. Under [D-Arg8]-inotocin, contraction amplitude was significantly reduced at 10, 30 and 100 nM (A) whilst area-under-the-curve (AUC) was significantly reduced at 100 nM (B). Following treatment with SR49059, amplitude of contraction and AUC were significantly reduced at 30 nM and 100 nM (C and D, respectively); $^{*}P<0.05$, $^{**}P<0.01$, $^{***}P<0.001$ (n = 5, one-way ANOVA, Tukey's post hoc analysis).

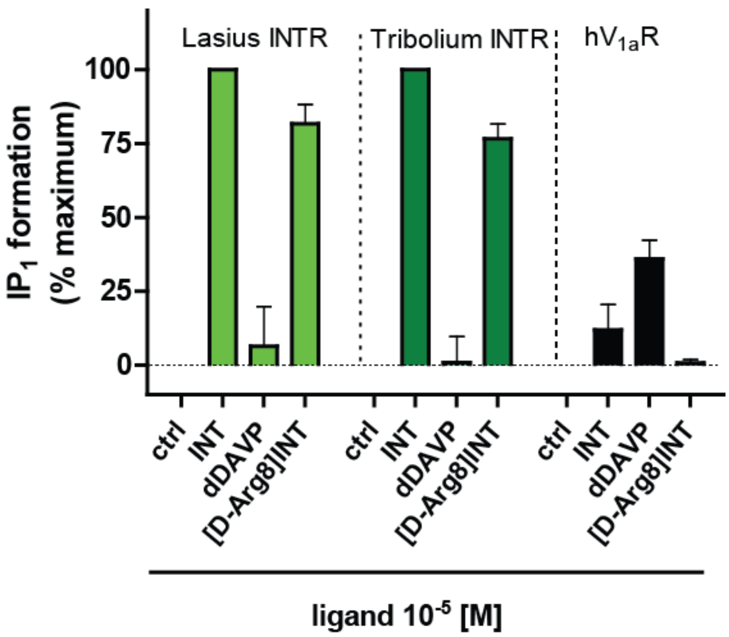

Supplementary Fig. 7. Desmopressin is inactive at the insect receptors. Functional second messenger quantification (IP_1 formation) on CHO cells transiently expressing inotocin receptors (INTR) of *L. niger* and *T. castaneum*, respectively, and HEK293 cells expressing human $V_{1a}R$. The effect of [D-Arg8]-inotocin was analysed in comparison to endogenous inotocin and desmopressin (1-desamino-8-D-arginine vasopressin, dDAVP), carrying also a D-aa analogue in position 8 (n = 3). Cells were treated with 10 µM of each peptide.

Supplementary References

1 Pitti, T. & Manoj, N. Molecular evolution of the neuropeptide S receptor. *PLoS One* **7**, e34046, (2012).
